# Author Correction: Forecasting the dissemination of antibiotic resistance genes across bacterial genomes

**DOI:** 10.1038/s41467-026-74295-3

**Published:** 2026-06-18

**Authors:** Morten O. A. Sommer, Christian Munck

**Affiliations:** The Novo Nordisk Foundation Center for Biosustainability, Technical University of Denmark, Lyngby, Denmark

Correction to: *Nature Communications* 10.1038/s41467-021-22757-1, published online 23 April 2021

In the original version of the article^1^, we described a statistical approach to identify putative mobilisation elements and other features associated with transfer of antibiotic resistance genes (ARGs) among bacterial clades to predict the potential future dissemination of known ARGs. We tested our predictions using a separate database with over 470,000 genomes from *Streptococcaceae*, *Staphylococcaceae* and *Enterobacteriaceae*, confirming 34 of 94 predicted mobilisations.

Godron et al.^2^ subsequently noted that 30 of the 34 transfer events identified in our confirmatory analysis likely arose from contaminant sequences rather than true host acquisitions. For example, in one *Staphylococcaceae* isolate (SRA: ERR212931) where we reported a *catI* resistance gene alongside the IS1 mobile element, their re-assembly indicated that the contig was derived from *Acinetobacter* contaminant DNA.

We recognize that contaminant DNA likely inflated the raw number of confirmed events in our original publication. However, the presence of contamination in the SRA database does not invalidate the central premise of our confirmation step: that some predicted ARG transfers are present in existing genomic data. Further discussion of these issues is available in our Reply^3^ to Godron et al.

These errors have not been corrected in either the PDF or HTML versions of the article.
