# Supplementary material for: Forecasting the dissemination of antibiotic resistance genes across bacterial genomes
Source: Nat Commun. 2021 Apr 23;12:2435. doi: 10.1038/s41467-021-22757-1 (PMC8065159; doi:10.1038/s41467-021-22757-1)
Supplement: Supplementary file 1 — Supplementary Information [file 41467_2021_22757_MOESM1_ESM.pdf]

Forecasting the dissemination of antibiotic resistance genes across bacterial genomes: Supplementary Information

Mostafa Ellabaan, Christian Munck,
Andreas Porse, Lejla Imamovic & Morten Sommer

March 17, 2021

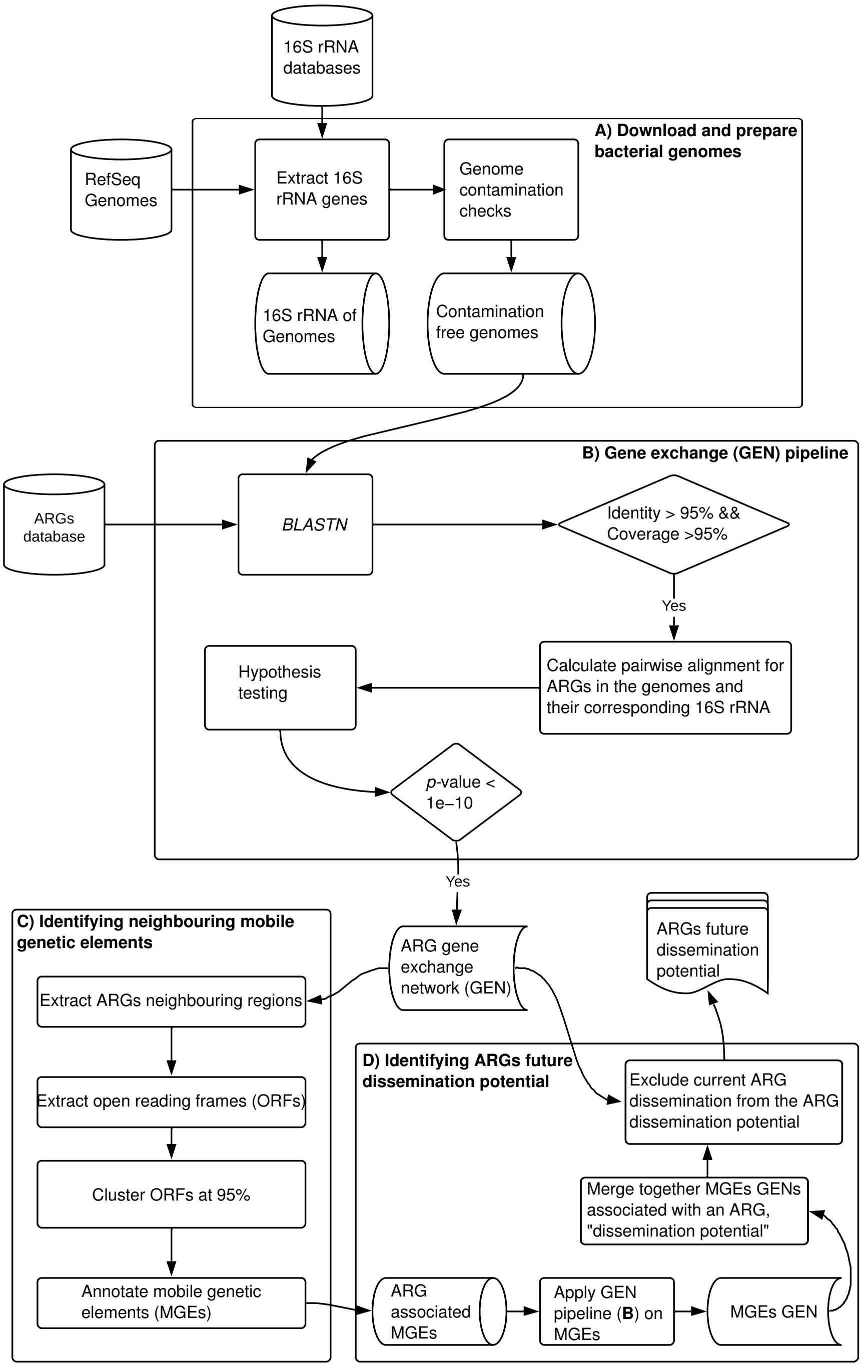

Supplementary Fig. 1: Summary of the computational framework of this study. A) Genome preparation and exclusion of contaminated genomes. B) Identification of ARG gene exchange network (GEN). C) Identification of ARG neighbouring mobile genetic elements. D) Identification of the future dissemination potential of ARGs based on the phylogenetic reach of their associated mobile genetic element (MGE).

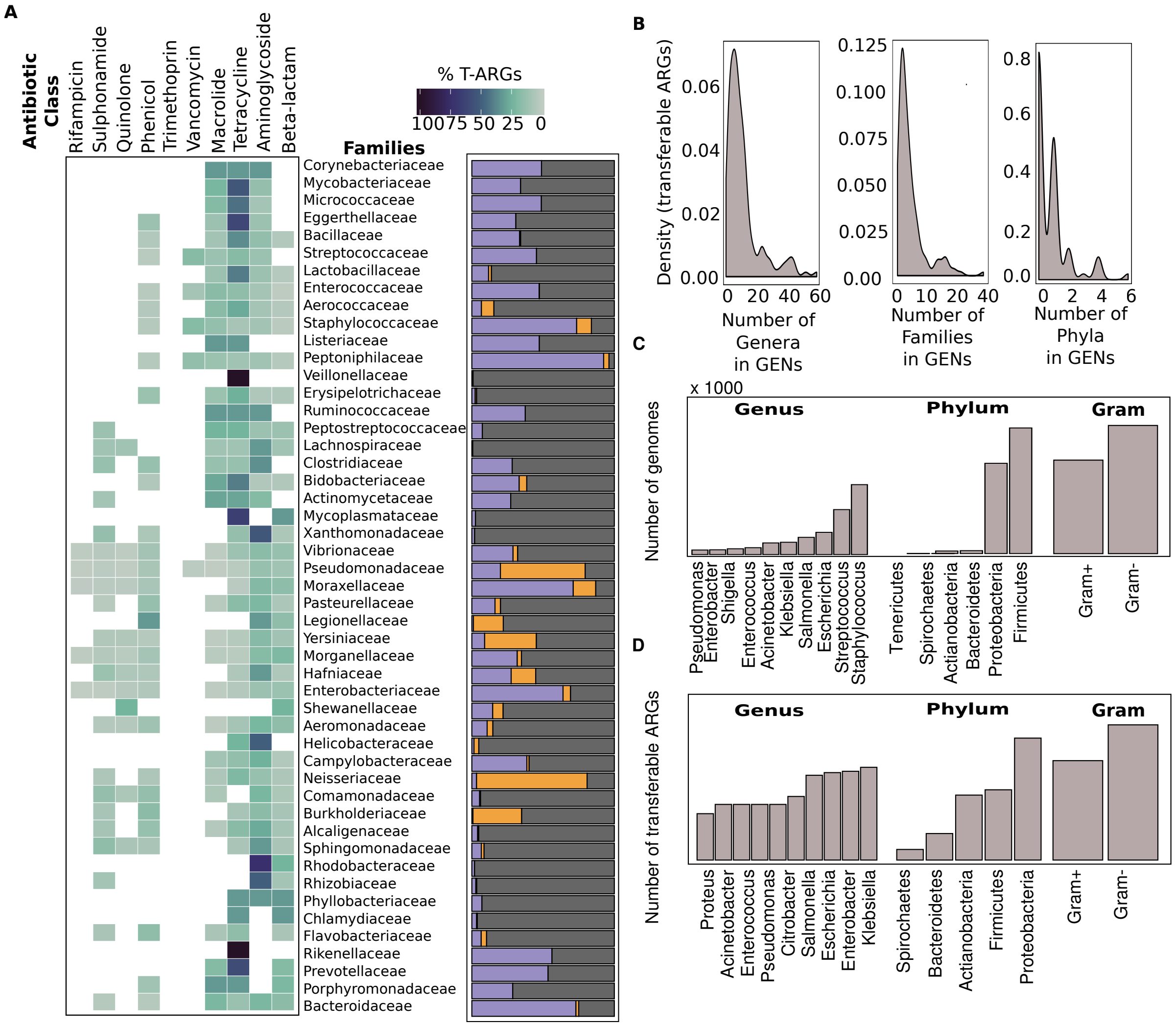

Supplementary Fig. 2: Resistance genes by classification. A) Heatmap of % transferable antibiotic resistance genes (T-ARGs) per antibiotic class for indicated families with percent of genomes in each family with transferable resistance (purple), nontransferable resistance (orange), no known resistance (gray). B) Distributions of antibiotic resistance genes (ARGs) based on GEN size measured as number of genera, families, and phyla. C) Top classifications (genera, phyla and gram group) based on number of genomes (in thousands) with T-ARGs that appeared in gene exchange networks. D) Number of T-ARGs in indicated groups. Source data are provided in Supplementary Data 3 and 14.

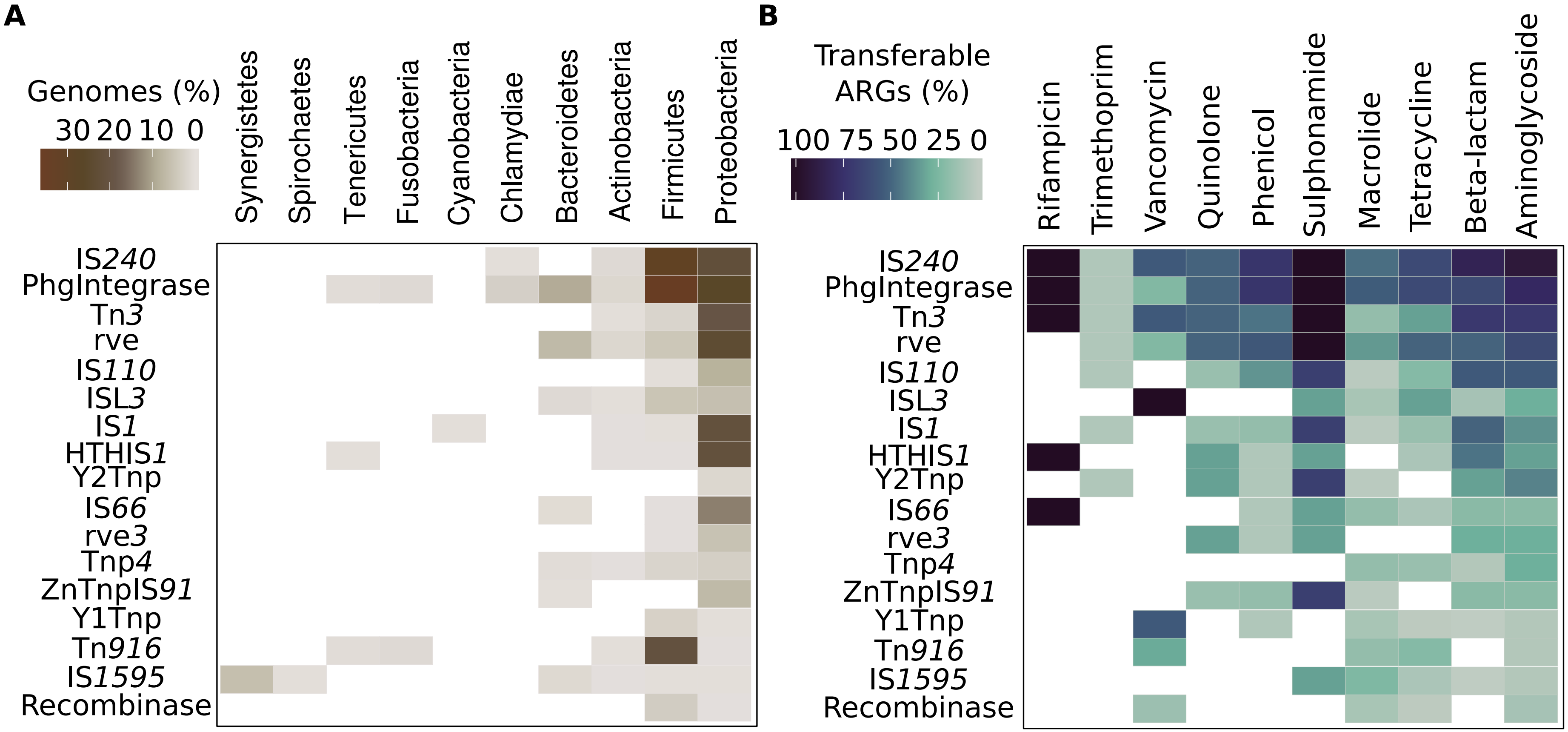

Supplementary Fig. 3: Abundance of antibiotic resistance genes (ARGs) and associated mobile genetic elements. A) Heatmap showing percentage of genomes in a phylum that had an observed mobile genetic element. B) Heatmap showing percentage of transferable ARGs by antibiotic class to which they confer resistance and associated mobile genetic element. Source data are provided in Supplementary Data 1, 5 and 14.

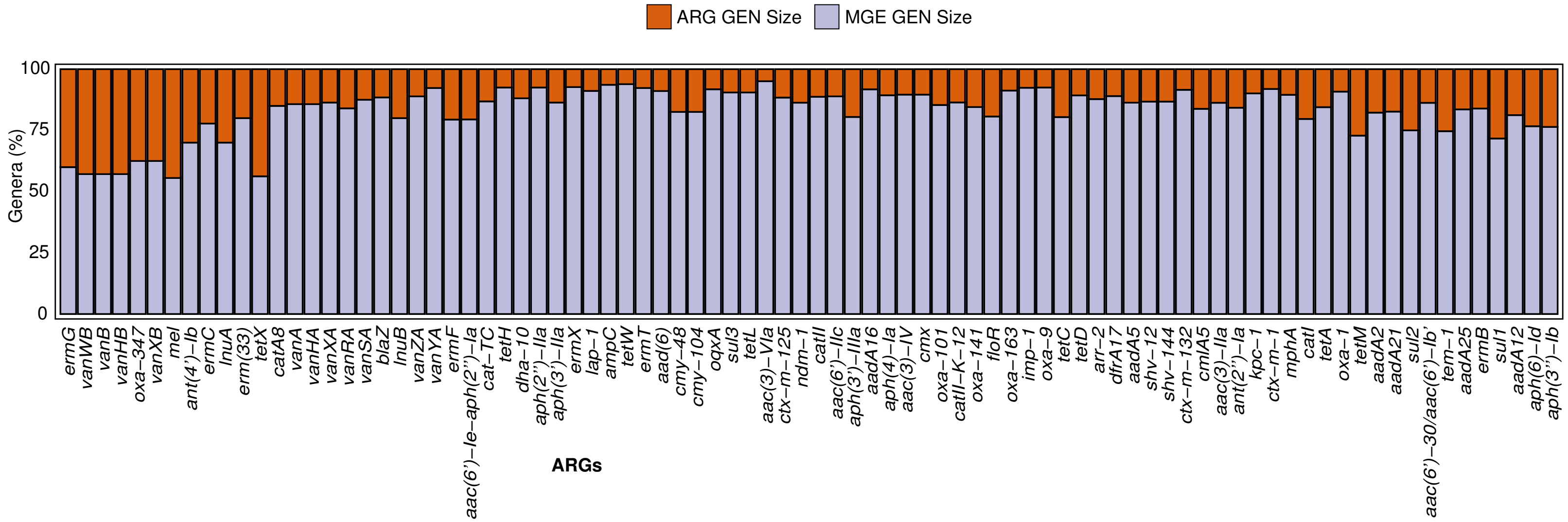

Supplementary Fig. 4: Current antibiotic resistance gene (ARG) gene exchange network (GEN) sizes (as % genera that include the indicated ARG) with associated MGE GEN size (as % genera that include an MGE near the ARG), showing the current and future dissemination potential of the indicated ARGs. ARGs are ascendingly sorted based on their associated MGE GEN size (or the future dissemination potential) from left to right. Source data are provided in Supplementary Data 1, 3, 5 and 14.

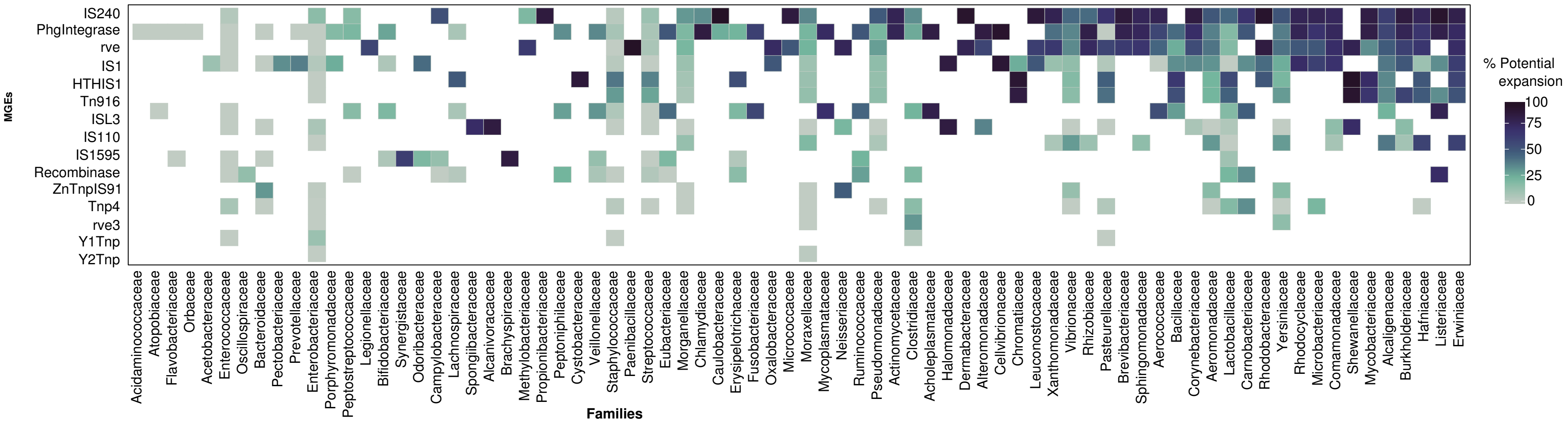

Supplementary Fig. 5: Contribution of mobile genetic elements (MGEs) in moving antibiotic resistance genes (ARGs) to bacteria, by family. Potential expansion is shown as percent of total ARGs analysed that could be carried by the indicated MGE to the indicated family. Source data are provided in Supplementary Data 3, 5 and 14.

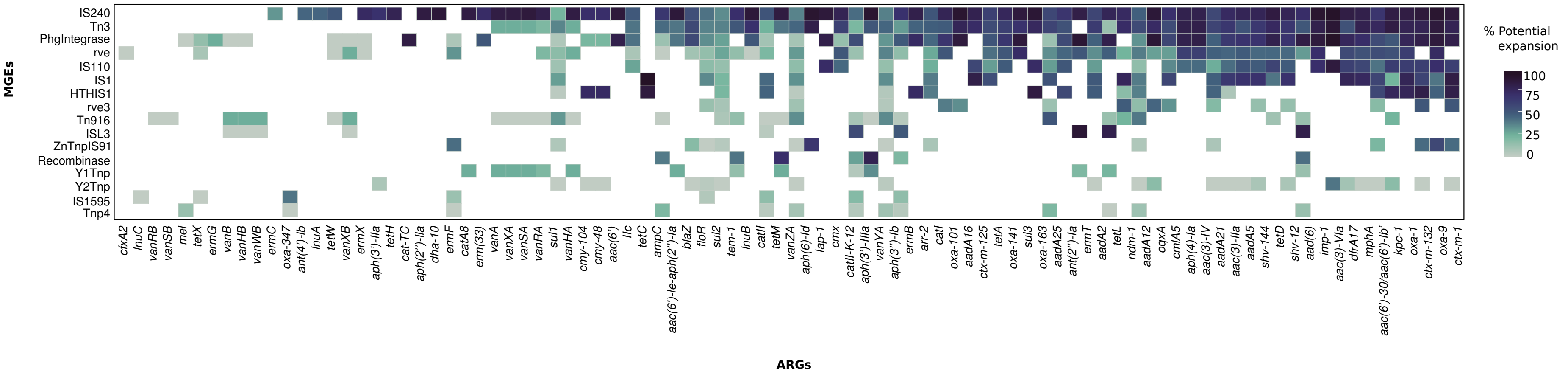

Supplementary Fig. 6: Contribution of mobile genetic elements (MGEs) to disseminating antibiotic resistance genes (ARGs), by cluster, to new bacterial families. Potential expansion is shown as percent of analysed bacterial families that could receive the indicated ARG carried by the indicated MGE. Source data are provided in Supplementary Data 1, 3 and 5.
