# Supplementary material for: Forecasting the dissemination of antibiotic resistance genes across bacterial genomes
Source: Nat Commun. 2021 Apr 23;12:2435. doi: 10.1038/s41467-021-22757-1 (PMC8065159; doi:10.1038/s41467-021-22757-1)
Supplement: Supplementary file 3 — Description of Additional Supplementary Files [file 41467_2021_22757_MOESM3_ESM.pdf]

Description of Additional Supplementary Files

File Name: Supplementary Data 1
Description: A comprehensive and manually curated database of ARGs.

File Name: Supplementary Data 2
Description: ARGs predicted to be horizontally transferred with high confidence. P-value calculated considering the left-tailed Mann-Whitney-Wilcoxon hypothesis testing in R (wilcox.test) and corrected using Bonferroni correction.

File Name: Supplementary Data 3
Description: Antibiotic resistance gene exchange networks mined from NCBI RefSeq genomes.

File Name: Supplementary Data 4
Description: A database of ARG-associated mobile genetic elements.

File Name: Supplementary Data 5
Description: ARG – associated MGEs gene exchange networks mined from NCBI RefSeq genomes.

File Name: Supplementary Data 6
Description: Ranking transferable MGEs based on the number of different ARGs they were associated with.

File Name: Supplementary Data 7
Description: Ranking transferable MGEs based on their phylogenetic reach.

File Name: Supplementary Data 8
Description: Number and percentage of transferable MGEs facilitating the dissemination of different antibiotic classes.

File Name: Supplementary Data 9
Description: Future hosts of ARGs: The difference between MGEs gene exchange networks and ARGs gene exchange network.

File Name: Supplementary Data 10
Description: ARGs dissemination potential (in terms of "genera").

File Name: Supplementary Data 11
Description: ARGs observed in SRA genomes of Streptococcaceae, Enterobacteriaceae, and Staphylococcaceae.

File Name: Supplementary Data 12

Description: Current dissemination versus future dissemination of ARGs (in terms of “species”)

File Name: Supplementary Data 13
Description: Experimental data testing of ARGs in*B. Subtilis.*

File Name: Supplementary Data 14
Description: RefSeq bacterial genomes and their phylogenetic information.
