# Supplementary material for: Forecasting the dissemination of antibiotic resistance genes across bacterial genomes
Source: Nat Commun. 2021 Apr 23;12:2435. doi: 10.1038/s41467-021-22757-1 (PMC8065159; doi:10.1038/s41467-021-22757-1)
Supplement: Supplementary file 18 — Reporting Summary [file 41467_2021_22757_MOESM18_ESM.pdf]

nature research

Corresponding author(s): Morten Sommer, Mostafa Ellabaan

Last updated by author(s): 26/2/2021

Reporting Summary

Nature Research wishes to improve the reproducibility of the work that we publish. This form provides structure for consistency and transparency in reporting. For further information on Nature Research policies, see our Editorial Policies and the Editorial Policy Checklist.

Statistics

For all statistical analyses, confirm that the following items are present in the figure legend, table legend, main text, or Methods section.

n/a	Confirmed	
☐	☒	The exact sample size (*n*) for each experimental group/condition, given as a discrete number and unit of measurement
☐	☒	A statement on whether measurements were taken from distinct samples or whether the same sample was measured repeatedly
☐	☒	The statistical test(s) used AND whether they are one- or two-sided<br>*Only common tests should be described solely by name; describe more complex techniques in the Methods section.*
☒	☐	A description of all covariates tested
☐	☒	A description of any assumptions or corrections, such as tests of normality and adjustment for multiple comparisons
☐	☒	A full description of the statistical parameters including central tendency (e.g. means) or other basic estimates (e.g. regression coefficient) AND variation (e.g. standard deviation) or associated estimates of uncertainty (e.g. confidence intervals)
☐	☒	For null hypothesis testing, the test statistic (e.g. *F*, *t*, *r*) with confidence intervals, effect sizes, degrees of freedom and *P* value noted<br>*Give P values as exact values whenever suitable.*
☒	☐	For Bayesian analysis, information on the choice of priors and Markov chain Monte Carlo settings
☒	☐	For hierarchical and complex designs, identification of the appropriate level for tests and full reporting of outcomes
☒	☐	Estimates of effect sizes (e.g. Cohen's *d*, Pearson's *r*), indicating how they were calculated

Our web collection on statistics for biologists contains articles on many of the points above.

Software and code

Policy information about availability of computer code

Data collection	wget, ftp and curl
Data analysis	cd-hit, NCBI BLASTN, EMBOSS Matcher, Mann-Whitney-Wilcoxon test in R (wilcox.test), single-cell assembler SPAdes

For manuscripts utilizing custom algorithms or software that are central to the research but not yet described in published literature, software must be made available to editors and reviewers. We strongly encourage code deposition in a community repository (e.g. GitHub). See the Nature Research guidelines for submitting code & software for further information.

Data

Policy information about availability of data

All manuscripts must include a data availability statement. This statement should provide the following information, where applicable:

- Accession codes, unique identifiers, or web links for publicly available datasets
- A list of figures that have associated raw data
- A description of any restrictions on data availability

All raw data used in this study are available in NCBI RefSeq and SRA data. We retrieved the data associated with whole-genome-sequenced bacterial genomes from both RefSeq and SRA. The numerical data underlying all figures are available in Supplementary Data 1-14. Source data are provided with this paper. All relevant data are available from the corresponding authors.

Field-specific reporting

Please select the one below that is the best fit for your research. If you are not sure, read the appropriate sections before making your selection.

- [x] Life sciences
- [ ] Behavioural & social sciences
- [ ] Ecological, evolutionary & environmental sciences

For a reference copy of the document with all sections, see nature.com/documents/nr-reporting-summary-flat.pdf

Life sciences study design

All studies must disclose on these points even when the disclosure is negative.

Sample size	The sample size was chosen based on previous experiments with similar methodologies and variablity outcomes in the literature [14]. 14 - Porse, A., Schou, T. S., Munck, C., Ellabaan, M. M. & Sommer, M. O. Biochemical mechanisms determine the functional compatibility of heterologous genes. Nature Communications (2018).
Data exclusions	We excluded contaminated genomes from our analysis.
Replication	Three replicates were made for the experimental procedures of antiboitic sensitivity of B. Subtilis carrying different ARGs. The result were always consistent across replicates and often survival, and thus ARG activity, would be validated across several concentrations of each antibiotic.
Randomization	We did not apply randomization to our study as we have already considered all the data on bacterial genomes deposited at NCBI RefSeq and NCBI SRA. We use statistical testing considering the whole data set deposited in the NCBI RefSeq to predict the future dissemination of resistance genes. The result was confirmed using whole bacterial genomes deposited at NCBI SRA but not at NCBI RefSeq.
Blinding	We did not apply blinding to our study as we have already considered all the data on bacterial genomes deposited at NCBI RefSeq and NCBI SRA. We use statistical testing considering the whole data set deposited in the NCBI RefSeq to predict the future dissemination of resistance genes. The result was confirmed using whole bacterial genomes deposited at NCBI SRA but not at NCBI RefSeq.

Reporting for specific materials, systems and methods

We require information from authors about some types of materials, experimental systems and methods used in many studies. Here, indicate whether each material, system or method listed is relevant to your study. If you are not sure if a list item applies to your research, read the appropriate section before selecting a response.

Materials & experimental systems

n/a	Involved in the study	
☒	☐	Antibodies
☒	☐	Eukaryotic cell lines
☒	☐	Palaeontology and archaeology
☒	☐	Animals and other organisms
☒	☐	Human research participants
☒	☐	Clinical data
☒	☐	Dual use research of concern

Methods

n/a	Involved in the study	
☒	☐	ChIP-seq
☒	☐	Flow cytometry
☒	☐	MRI-based neuroimaging
